# Accumulation of heavy metals and human health risk assessment via the consumption of freshwater fish *Mastacembelus armatus* inhabiting, thermal power plant effluent loaded canal

**DOI:** 10.1186/s40064-016-2471-3

**Published:** 2016-06-18

**Authors:** Mehjbeen Javed, Nazura Usmani

**Affiliations:** Aquatic Toxicology Research Laboratory, Department of Zoology, Aligarh Muslim University, Aligarh, Uttar Pradesh 202002 India

**Keywords:** Heavy metals, Bioaccumulation, Dietary intake, Target hazard quotient, Carcinogenic risk, Human health risk

## Abstract

Bioaccumulation of six heavy metals (Mn, Fe, Co, Ni, Cu, Zn) in the muscle of highly consumed fish species (*Mastacembelus armatus*) were measured using atomic absorption spectrometer. Fe (213.29 mg/kg dry weight) concentration was the most, followed by Zn (186.19 mg/kg dry weight), Ni (58.98 mg/kg dry weight), Cu (41.36 mg/kg dry weight), Co (9.06 mg/kg dry weight) and Mn (9.03 mg/kg dry weight). Estimated daily intake of heavy metals was calculated by mean fish consumption rate 19.5 × 10^−3^ kg/day, on the basis of a calculation of the amount of fish consumed by adult individuals (male and female). The studied fish species pose non carcinogenic risk for Co and Ni [target hazard quotient (THQ) > 1] only. Hazard index (HI) was high. Carcinogenic risk (TR) posed by this fish for male and female was 3.43 × 10^−3^ and 3.91 × 10^−3^, respectively for Ni (the carcinogenic potency slope factor was available for Ni only). The study is an alert indicating that inhabitants who consume these fishes (particularly females) were at risk of Co and Ni toxicity. In India recommended guidelines have yet not been established for these heavy metals, which is essential for setting of toxicological standards.

**Electronic supplementary material:**

The online version of this article (doi:10.1186/s40064-016-2471-3) contains supplementary material, which is available to authorized users.

## Background

Heavy metals are non-biodegradable and persistent and are known to cause deleterious effects on animal and human health (Davydova 2005; Javed and Usmani 2011, 2012). Both acute and prolonged exposures to heavy metals cause various diseases (Jarup 2003; Javed and Usmani 2013a, 2015). Dietary intake of toxic elements is the main route of exposure for most people (Calderon et al. 2003; Powers et al. 2003). Owing to industrialization, heavy metal pollution of aquatic ecosystems has become topic of concern worldwide. Fishes are on top of aquatic food chain and hence accumulate significant amount of heavy metals (Javed and Usmani 2013b, 2015) and become the source of heavy metal for consumers. Primarily fishes are consumed as they are one of the best sources of protein and polyunsaturated fatty acids (PUFA). According to American Heart Association (AHA) fishes are recommended twice a week to the adults with no history of heart attack (Kris-Etherton et al. 2002).

A comprehensive regional survey of heavy metals in food and an assessment of their risk to the general population is lacking. In the present study, the concentrations of Mn, Fe, Co, Ni, Cu and Zn in freshwater fish *Mastacembelus armatus* inhabiting the heavy metal laden Kasimpur canal were determined. The potential risks (non-carcinogenic and carcinogenic) of heavy metals by consuming contaminated fish for adult male and female individuals were also estimated.

## Methods

### Sample collection and preparation

*Mastacembelus armatus* (15 samples) were collected from Kasimpur canal (28. 013°N and 78.126°E), district Aligarh, India. Fishes were captured with the help of professional local fisherman during the month of May, 2015. The fishes were captured with the help of cast net. They were washed with distilled water and kept in ice box and then transferred to the laboratory for further treatment. All the experiments were approved by the institutional ethical committee of Department of Zoology, Aligarh Muslim University, Aligarh, India. *M. armatus* is highly consumed by large mass of population of Aligarh region. Fish muscle was excised and dried in an oven at ±60 °C for 5–6 h. It was then powdered in pestle and mortar.

### Estimation of heavy metals

Heavy metals namely Mn, Fe, Co, Ni, Cu and Zn were assessed in the muscle of *M. armatus*. Dried tissue (1 g) was digested in analytical grade HNO_3_:HClO_4_ (4:1). After digestion the samples volume were raised up to the mark (50 ml), mixed thoroughly and used for the estimation of heavy metals using Atomic Absorption Spectrophotometer (Perkin Elmer, Analyst A 800) (Javed et al. 2015).

Instrument calibration standards were made by diluting the standard (1000 ppm) supplied by Wako Pure Chemical Industry Ltd., Japan. Analytical blanks were run in the same way as the samples and concentrations were determined using standard solutions prepared in the same acid matrix. The accuracy of the applied analytical procedure was tested using the certified reference material Dorm-2 (dogfish muscle, National Research Council, Canada) for investigated metals. Replicate analyses of these reference materials gave good accuracy, with recovery rates for metals between 97 and 104 % for fish (Additional file 1: Table 1) provided as supplementary material.

### Calculation of health risk assessment for fish consumption

The values of heavy metal accumulation in muscle were used to calculate the estimated daily intake of metals (EDI), target hazard quotients (THQ), hazard index (HI) and target cancer risk (TR) separately for adult male and female individuals.

### Estimated daily intake of metals (EDI)

EDI is measured in (mg/kg body-weight/day) (Song et al. 2009).

$$EDI = \frac{Mc \times IR}{{Bw \times 10^{ - 3} }}$$

where, Mc is the metal concentration in the fish muscle (mg/kg dry weight). IR is the ingestion rate, which is taken as 19.5 × 10^−3^ kg/day as taken by previous studies (Little et al. 2002; Speedy 2003) and this consumption rate was used in health-risk assessment. The assumption of an adult ingestion rate of fish over a lifetime is a high estimate of actual fish consumption. BW is an average body weight of Indian male which is taken as 57 kg and that of female as 50 kg (Shukla et al. 2002).

### Target Hazard Quotient (THQ)

To assess the human health risk from consuming the fish contaminated with heavy metals, the target hazard quotient (THQ) was calculated as per US EPA Region III Risk-Based Concentration Table [USEPA (United States Environmental Protection Agency) 2011]. The THQ is an estimate of the non-carcinogenic risk level due to pollutant exposure and calculated by the following equation:

$$THQ = \frac{{\left( {M_{c} \times {\text{IR }} \times 10^{ - 3} \times {\text{EF }} \times {\text{ED}}} \right)}}{{\left( {RfD \times BW \times ATn} \right)}}$$

where, THQ is non-carcinogenic risk and is dimensionless. EF is the exposure frequency (365 days/year). ED is the exposure duration (67 years) (life expectancy of male = 65 years approx. and for females is 68 years approx. in India, therefore an average of two extremes have been taken). RfD is the reference dose of individual metal (mg/kg/day) [USEPA (United States Environmental Protection Agency) 2012] (Table 1). ATn is the averaging time for non-carcinogens (365 days/year × ED) [USEPA (United States Environmental Protection Agency) 2011].

**Table 1:** Reference dose and carcinogenic potency slope factor, oral

Heavy metals	RfD (mg/kg/day)	CPSo (mg/kg bw-day^−1^)
Mn	1.4 × 10^−1^	–
Fe	7.0 × 10^−1^	–
Co	3.0 × 10^−4^	–
Ni	2.0 × 10^−2^	1.7
Cu	4.0 × 10^−2^	–
Zn	3.0 × 10^−1^	–

Data taken from USEPA 2011, 2012 respectively

### Hazard Index (HI)

To assess the overall potential health risk posed by more than one metal, THQ of every metal is summed up and is known as hazard index (HI). The HI can be calculated by the sum of the target hazard quotients of each metal [USEPA (United States Environmental Protection Agency) 2011].

$${\text{HI}} = {\text{THQMn}} + {\text{THQFe}} + {\text{THQCo}} + {\text{THQNi}} + {\text{THQCu}} + {\text{THQZn}}$$

### Target Cancer Risk (TR)

Target cancer risk (TR) is used to indicate the carcinogenic risk. The method which is used to estimate TR is provided in USEPA Region III Risk-Based Concentration Table [USEPA (United States Environmental Protection Agency) 2011]. It is dimensionless. TR was calculated by the following equation:

$$TR = \frac{{M_{c} \times {\text{IR }} \times 10^{ - 3} \times {\text{CPSo }} \times {\text{EF }} \times {\text{ED}}}}{{{\text{BW }} \times {\text{ATc}}}}$$

where M_C_, IR, EF, ED, BW are already explained above. CPSo is the carcinogenic potency slope, oral (mg/kg bw-day^−1^). ATc is the averaging time for carcinogens (365 days/year × 67 years), since in India the average life expectancy for males is 65 years (approx.) and for females is 68 years (approx.), therefore an average of two extremes have been taken for carcinogenic averaging time (http://countryeconomy.com/demography/life-expectancy/india).

Since Mn, Fe, Co, Cu and Zn do not cause any carcinogenic effects as their CPSo have yet not been established (USEPA 2012) so, TR value for intake of only Ni was calculated to show the carcinogenic risk. Its slope factor oral (CPSo) is the calculated slope as fixed by USEPA (2012) is given in Table 1.

There are certain assumptions which should be taken while evaluating the THQ for human health risk which are as follows:

- Ingested dose of pollutant is equal to the absorbed dose [USEPA (United States Environmental Protection Agency) 1989].
- Cooking has no effect on pollutants (Forti et al. 2011).

## Results and discussion

### Bioaccumulation of heavy metals in muscle of fish

The concentration of different heavy metals (Mn, Fe, Co, Ni, Cu, Zn) in the edible part (muscle) of fishes collected from Kasimpur canal is given in Table 2. Fe (213.29 mg/kg dry weight) accumulated the most, followed by Zn (186.19 mg/kg d.w.), Ni (58.98 mg/kg d.w.), Cu (41.36 mg/kg d.w.), Co (9.06 mg/kg d.w.) and Mn (9.03 mg/kg d.w.). Accumulations of heavy metals in tissues of other fishes have also been reported from the region (Javed et al. 2016a, b).

**Table 2:** Heavy metal concentration (mg/kg. d.w) in muscle of *M. armatus*

Heavy metals	Muscle	Recommended limits (ppm)
Mn	9.03 ± 0.04	1.0 (FAO/WHO 1989)
Fe	213.29 ± 0.48	100 (FAO/WHO 1989)
Co	9.06 ± 0.04	**
Ni	58.98 ± 0.06	70–80 (USFDA 1993)
Cu	41.36 ± 0.38	30 (FAO/WHO 1983)
Zn	186.19 ± 0.12	100 (FAO/WHO 1989)

All values are given as mean ± SEM (n = 15); ** No guidelines

In the current study muscle was particularly selected for heavy metal analysis because it is the only edible tissue and thus concentration of toxicants in it was of concern. Accumulation of Mn, Fe, Cu and Zn was higher than the recommended guidelines (FAO/WHO 1983, 1989; USFDA 1993). Therefore, human health risk assessment was carried out to estimate the risk posed by these metals.

### Human health risk assessment

Estimated daily intake (EDI), Target hazard quotient (THQ), Hazard index (HI) and Target cancer risk (TR) values of metals via consumption of fish *M. armatus* are given in Table 3.

**Table 3:** Estimated daily intake (EDI), Target hazard quotient (THQ), Hazard index (HI) and Target cancer risk (TR) values of metals via consumption of fish *M. armatus*

Heavy metals	EDI (mg/kg body-weight/day)		THQ		HI		TR	
	Male	Female	Male	Female	Male	Female	Male	Female
Mn	3.08 × 10^−3^	3.5 × 10^−3^	0.022	0.025	12.02	13.71	–	–
Fe	7.29 × 10^−2^	8.31 × 10^−2^	0.104	0.118			–	–
Co	3.09 × 10^−3^	3.53 × 10^−3^	10.33	11.77			–	–
Ni	2.02 × 10^−2^	2.30 × 10^−2^	1.008	1.15			3.43 × 10^−3^	3.91 × 10^−3^
Cu	1.41 × 10^−2^	1.61 × 10^−2^	0.353	0.403			–	–
Zn	6.37 × 10^−2^	7.26 × 10^−2^	0.212	0.242			–	–

EDI values were many folds higher than the respective reference doses.

THQ was highest for Co followed by Ni > Cu > Zn > Fe > Mn for both male and female individuals.

High HI value was estimated for both females (13.71) and males (12.02).

Females (3.91 × 10^−3^) were more prone to carcinogenic risk (TR) than males (3.43 × 10^−3^) (for Ni).

According to Environmental Protection Agency (EPA) human health risk assessment is defined as the process to estimate the nature and probability of adverse health effects in humans exposed to chemicals in contaminated environmental media, now or in the future (http://www.epa.gov/risk_assessment/health-risk.htm). Risk assessment for heavy metals is estimated using parameters viz estimated daily intake (EDI), target hazard quotient (THQ), hazard index (HI) and target cancer risk (TR). These parameters for risk assessment were introduced by EPA in the United States for the estimation of potential health risk caused by any chemical contaminant over prolonged exposure [USEPA (United States Environmental Protection Agency) 1989]. These parameters depend not only on intake amount of contaminant but also deal with exposure frequency and duration, average body weight and oral reference dose (RfD). THQ is a dimensionless quantity and is a ratio of concentration of heavy metal content in the food item to its RfD, weighed by duration and frequency of exposure, intake amount and body weight (Harmanescu et al. 2011). THQ should not exceed 1, else it indicates to pose potential non carcinogenic risks to exposed population (Abdou and Hassan 2014; Harmanescu et al. 2011; Jovic and Stankovic 2014). It should also be noted that THQ is not a measure of risk but it reflects the level of concern (Harmanescu et al. 2011; Khan et al. 2009).

In the present study Co and Ni both show THQ values >1. Moreover, the THQ values for all concerned heavy metals were comparatively higher in females than males. This could be due to the differences in average weight and lifespan hence the risk assessment parameters namely EDI, THQ and TR were calculated separately for adult male and female individuals. According to New York State Department of Health [NYSDOH (New York State Department of Health) 2007], if the ratio of EDI of heavy metal to its RfD was equal to or less than the RfD then the risk will be minimum. But if it is >1–5 times the RfD then risk will be low, if >5–10 times the RfD then risk will be moderate, however, if >10 times the RfD then the risk will be high. Ratio obtained for Mn, was approximately two folds higher for Ni. Cu and Zn around seven folds and for Co several thousand times higher than their RfD, indicating potential health hazard to the public. Among the concerned heavy metals it is Co whose permissible limit has yet not been established by any agency in the world and as well as India.

THQ deal with individual heavy metal only, but generally food items contain more than one heavy metal as already seen in the case of fish muscle, six heavy metals were detected. So it becomes mandatory to calculate hazard index (HI). It is the numerical sum of all the THQs calculated for the fish fillet. Like THQ it should also not exceed 1 (Islam et al. 2014; Zodape 2014), if does then it is an alarm for public health concern. Adult females were found to be more prone to heavy metal risk than males (Vahter et al. 2002).

Among these concerned heavy metals Cr and Ni are mentioned in the list of potent carcinogens [USEPA (United States Environmental Protection Agency) 2012]. Cr was not detected hence target carcinogenic risk (TR) was calculated for Ni only. According to New York State Department of Health [NYSDOH (New York State Department of Health) 2007] the TR categories are described as, if TR ≤ 10^−6^ = Low; 10^−4^ to 10^−3^ = moderate; 10^−3^ to 10^−1^ = high; ≥10^−1^ = very high. In the study Ni shows high cancer risk to the exposed population. Like THQ the estimated lifetime cancer risk (TR) is also not a specific estimate of expected cancers. Rather, it is apparently an upper limit of the probability that the individuals may have cancer sometime his/her lifetime following exposure to that toxicant [NYSDOH (New York State Department of Health) 2007].

There are limits of intake even for the essential metals. Studies have shown that high intake of Fe and Mn is responsible for the deposition of iron oxides as has been reported in case of Parkinson’s disease [FDA (Food and Drug Agency) 2001; Powers et al. 2003]. Mn is essential element for both animals and plants and its deficiency results in severe skeletal and reproductive abnormalities in mammals (Sivaperumal et al. 2007). Co is essential for human health as it forms part of Vitamin B_12_ and around 0.16–1.0 mg/kg body weight is given to treat anemia [Agency for Toxic Substances and Disease Registry (ATSDR) 2004]. Short term exposure of rats to high doses of Co in food resulted in adverse health effects on blood, liver, kidney and heart [Agency for Toxic Substances and Disease Registry (ATSDR) 2004]. Based on the animal data of acute and chronic exposure to Co, the International Agency for Research on Cancer (IARC) has determined that it is possibly carcinogenic to humans [Agency for Toxic Substances and Disease Registry (ATSDR) 2004]. Excess of Cu is also found to be associated with liver damage. Excess of Zn show adverse nutrient interactions with Cu which means that high Zn concentration or intake around (50 mg/day) over a period of weeks can interfere with the availability of Cu to the body (King and Cousins 2006; Powers et al. 2003). According to King and Cousins (2006) high intake of Zn induces production of Cu binding proteins (metallothionein) in intestine which traps Cu within intestinal cells and prevents its systemic absorption. In addition to this excess Zn reduces immune function and the levels of high density lipoprotein (HDL) [FDA (Food and Drug Agency) 2001]. Ni normally occurs at very low levels in the environment and it may cause deleterious effects on pulmonary, like lung inflammation, fibrosis, emphysema and tumors (Forti et al. 2011).

## Conclusion

It may therefore be concluded that *M. armatus* in the study undertaken is not acceptable for either human consumption or their use in animal feeds. Its use is limited by Co and Ni. In addition to protein, fishes also serve as rich source of poly unsaturated fatty acids (PUFA) and so they are highly recommended in diet. It is a popular food fish both in urban and rural areas and India is one of the largest contributors of fish fillet in International markets. Therefore fishes if dwelling in contaminated waters should be consumed with caution lest it may cause carcinogenic and non-carcinogenic risks to the exposed population. This study contributes significant data to the various agencies of India in particular and other agencies like United States Environmental Protection Agency (USEPA), Federal Environmental Protection Agency (FEPA) etc. in general which work for the development of toxicological standards.

## Additional file

10.1186/s40064-016-2471-7 Concentrations of metals found in Standard Reference Material DORM-2 (dogfish muscle) from the National Research Council, Canada (all data as mean ± standard deviation, in mg kg^−1^ dry weight).
