# Supplementary material for: Accumulation of heavy metals and human health risk assessment via the consumption of freshwater fish Mastacembelus armatus inhabiting, thermal power plant effluent loaded canal
Source: Springerplus. 2016 Jun 18;5(1):776. doi: 10.1186/s40064-016-2471-3 (PMC4912512; doi:10.1186/s40064-016-2471-3)
Supplement: Supplementary file 1 — 10.1186/s40064-016-2471-7 Concentrations of metals found in Standard Reference Material DORM-2 (dogfish muscle) from the National Research Council, Canada (all data as mean ± standard deviation, in mg kg−1 dry weight). [file 40064_2016_2471_MOESM1_ESM.docx]

**Table 1. Concentrations of metals found in Standard Reference Material DORM-2 (dogfish muscle) from the National Research Council, Canada (all data as means ± standard deviation, in mg kg^-1^ dry weight).**

| **Metals** | **Certified ± SD** | **Observed ± SD** | **Recovery (%)** |
| --- | --- | --- | --- |
| Mn | 3.66 ± 0.236 | 3.574 ± 0.485 | 97.7 |
| Fe | 142 ± 9.45 | 143.096 ± 8.573 | 100.8 |
| Co | 0.065 ± 0.005 | 0.068 ± 0.007 | 104.6 |
| Ni | 19.4 ± 2.394 | 20.163 ± 1.846 | 103.9 |
| Cu | 2.34 ± 0.18 | 2.452 ± 0.115 | 104.8 |
| Zn | 26.6 ± 0.896 | 25.846 ± 1.967 | 97.2 |
